# Impact of the coronavirus disease pandemic on cancer care in Croatia: a multicentre cross-sectional study

**DOI:** 10.3332/ecancer.2021.1263

**Published:** 2021-07-07

**Authors:** Renata Kelemenic-Drazin, Anuska Budisavljevic, Natalija Dedic Plavetic, Iva Kardum Fucak, Tajana Silovski, Vesna Telesmanic Dobric, Mario Nalbani, Zvonimir Curic, Zvjezdana Boric-Mikez, Tatjana Ladenhauser, Dragan Trivanovic, Zeljko Vojnovic, Ilijan Tomas, Stjepko Plestina

**Affiliations:** 1 Department of Haematology, Oncology and Clinical Immunology, General Hospital Varazdin, I. Mestrovica 1, 42000, Varazdin, Croatia; 2 Department of Haematology and Oncology, General Hospital Pula, Zagrebacka 30, 52100, Pula, Croatia; 3 Department of Oncology, University Hospital Centre Zagreb, Kispaticeva 12, 10000, Zagreb, Croatia; 4 Department of Gastroenterology, Haematology and Oncology, General Hospital Dr. Tomislav Bardek Koprivnica, Ulica doktora Zeljka Selingera bb, 48000, Koprivnica, Croatia; 5 Department of Oncology and Nuclear Medicine, General Hospital Zadar, Ul. Boze Pericica 5, 23000, Zadar, Croatia; 6 Department of Oncology, General Hospital Dubrovnik, Dr. Roka Misetica 2, 20000, Dubrovnik, Croatia; 7 Department of Haematology and Oncology, General Hospital Dr. Josip Bencevic Slavonski Brod, Ul. Andrije Stampara, 35000, Slavonski Brod, Croatia; 8 Department of Oncology, University Hospital Centre Osijek, Ul. Josipa Huttlera 4, 31000, Osijek, Croatia

**Keywords:** cancer care, coronavirus, COVID-19, pandemic, patients' perspective

## Abstract

**Purpose:**

The coronavirus disease (COVID-19) pandemic has greatly affected the oncology community worldwide. Lockdowns, an epidemiological measure, have made it difficult for oncologists to provide care. In this study, we analysed the impact of the COVID-19 pandemic on Croatian cancer care.

**Methods:**

This was a multicentre cross-sectional observational study of 422 patients who received systemic oncology therapy during the pandemic. The patients completed a survey to capture their views on the impact of the COVID-19 pandemic on their cancer care. Univariate descriptive and bivariate analyses were performed to analyse the relationship between the patients’ perspective on the impact of the COVID-19 pandemic on cancer care and the quality of Croatian cancer care and their clinical and sociodemographic data.

**Results:**

Discontinuation or change in cancer treatment during the COVID-19 pandemic was observed in 10.2% of cases. Most did not change their place of treatment owing to the lockdown (97.6%). 14.7% of the patients felt that the quality of cancer care received had changed during the pandemic.

**Conclusions:**

In the first few months of the pandemic, Croatia had a favourable epidemiological situation. However, 25% of patients with cancer reported that the pandemic affected cancer treatment and the quality of cancer care.

## Background

The coronavirus disease (COVID-19) pandemic has greatly affected the oncology community and the wider health care system.

The first report on COVID-19 in patients with cancer was published in *The Lancet Oncology* in February 2020 [1] and suggests that cancer patients have a higher risk of COVID-19 and a poorer prognosis than non-cancer patients. Although initial opinions were that there was insufficient evidence to establish an association between cancer and COVID-19 [2, 3], new findings suggest that the presence of cancer significantly affects the mortality rate in patients with COVID-19 [4, 5].

To prevent the spread of the infection, the lockdown has been introduced. Therefore, oncology patients may not receive all of the necessary medical services, from hospital arrival to adequate systemic or local oncology therapy. This problem has become evident in Croatia, wherein 4.1 million inhabitants live in 20 counties and the capital, Zagreb. Seven of the 20 counties (with 18% of the total population) do not have organised cancer care facilities, and oncology patients in these counties are forced to travel to other counties daily to receive appropriate oncology treatment [6–8]. That could interrupt the continuity of oncology care and increase the risk of inadequate and delayed care during the COVID-19 pandemic and ultimately lead to poorer treatment outcomes [9, 10].

On 25 February 2020, the first case of COVID-19 in Croatia was reported in Zagreb [8]. The World Health Organization officially declared COVID-19 as a global pandemic on 11 March 2020 and called on governments to take urgent action [11].

By 24 May 2020 (date of completion of the current study), of the 2,244 people with COVID-19 in Croatia, 2,027 had recovered, 99 had died and 118 were still receiving treatment [8]. Considering these numbers compared with the 1 million inhabitants in Croatia, by the end of the study, 549 people were infected, and 24 patients had died. According to the number of deaths per million inhabitants, Croatia was among the five best-positioned countries in Europe [12–14].

This study aimed to determine the impact of the COVID-19 pandemic on cancer care and treatment in Croatia, as assessed by the patients that received treatment. We also investigated whether patients experienced a change in oncology treatment (change in treatment protocol or schedule), discontinuation of treatment or change in the treatment site. Furthermore, we also assessed whether the participants perceived that the quality of cancer care received during the pandemic had changed.

## Methods

### Study design and participants

An academic cross-sectional multicentre observational study was conducted between 20 April and 24 May 2020 (in the second and third months of the pandemic in Croatia), in eight oncology centres in Croatia: two university hospitals (UH) and six general/county hospitals (GH). This study included 422 patients and was initiated and conducted by the Council of Institutions of the Croatian Society for Medical Oncology, a collaborating society of the European Society for Medical Oncology.

The patients included in the study were older than 18 years, had already started systemic oncology treatment before 25 February 2020 (date of the first patient with COVID-19 reported in Croatia), were newly diagnosed with cancer and received oncology treatment during the pandemic. The study included patients with different stages of malignancy. The study included patients with early-stage cancers, whether treated with adjuvant or neoadjuvant therapy, as well as patients with disseminated malignancies treated in a metastatic, palliative setting.

We used a self-reported questionnaire entitled ‘The impact of the COVID-19 pandemic on the treatment of cancer patients in the Republic of Croatia’. Content validity of the survey was assessed by presenting it to a group of five oncologists. The obtained final version of the survey was further piloted on a sample of patients with cancer in different institutions (*n* = 20) to ensure that all the items used were clear and understandable. This questionnaire was approved by the Ethics Committee of the hospitals where it was conducted. All participants provided written informed consent for voluntarily participating in the study. No personal data were collected.

The questions in the first part of the survey collected data on the sociodemographic characteristics of patients, treating institution (UH or GH) and the distance the hospital was from the *patient’s* home. We divided patients into three groups according to the distance to the hospital (less than 50 km, 50–100 km, more than 100 km), education level and household income. Using data from the Croatian Central Bureau of Statistics [15], we divided patients into groups according to income. Patients with an average level of annual household income were included in the medium-income group, while the other patients were included in the low- or high-income groups (depending on whether their incomes were below or above the average level of annual household income in Croatia). Regarding the education level, we divided patients into the elementary school group (basic or primary), upper secondary school group (high school) and tertiary education group (undergraduate or postgraduate) [7]. We also divided patients according to their marital status (unmarried, divorced, widowed, in partnership (married or in a *de facto* relationship)).

The second part of the survey focussed on collecting data on the patients’ cancer and included questions on the cancer site, stage of disease (non-metastatic or metastatic cancer), duration of cancer treatment (less than 6 months, 6–12 months or longer than 12 months) and type of cancer therapy (chemotherapy, targeted/immunotherapy or a combination of different types of therapies: chemotherapy and targeted therapy, hormonal therapy and targeted therapy, immunotherapy and chemotherapy). Regarding the cancer site, patients were grouped as having breast cancer, gastrointestinal cancer (colorectal, pancreatic, gastric, oesophagus, *gallbladder* and b*ile duct cancer*), genitourinary cancer (prostate, kidney and bladder cancer), lung cancer, other sites (sarcomas, melanomas and head and neck cancer) and gynaecological cancer (ovarian and endometrial cancer).

In the third part of the survey, we determined whether, entirely due to the pandemic, the patients’ cancer treatment discontinued or changed and whether they perceived a change in the quality of cancer care received (quality of cancer treatment or quality of cancer diagnosis). The change in treatment referred to a change in the place of treatment (referral of the patient to the nearest oncology centre) or a change in the treatment schedule (such as replacing 2-week or 1-week cycles of therapy with 3-week ones) or type of therapy (such as changing parenteral therapies to oral ones). This did not apply to patients for whom treatment was discontinued or changed due to the progression of malignancy. The total number of completed questionnaires was 422.

### Statistical analysis

Univariate descriptive analyses were carried out to describe the data. The Kolmogorov–Smirnov test was used to test the normality of data distribution. For descriptive analysis, continuous variables were presented as the median with an interquartile range (IQR). Categorical variables were presented as a number (%). A bivariate analysis, chi-square test for categorical data and Fisher’s exact test were used to study the relationship between patients’ perspectives on the impact of the COVID-19 pandemic on oncology treatment as well as the relationship between changes in the quality of cancer treatment and diagnostics, and other clinical and sociodemographic data. During the *post hoc* analysis, Bonferroni’s correction was used to control for Type I error.

All statistical analyses were carried out using SPSS Statistics, version 20.0. A two-sided *p*-value of less than 0.05 was considered to indicate statistical significance.

## Results

### Demographic and clinical characteristics

The demographic and clinical features of this cohort are summarised in Table 1. The median patient age was 60.0 years. Most of the respondents were female (*n* = 283, 67.1%), and the majority of the patients had low- (*n* = 191, 45.3%) and medium-income levels (*n* = 164, 38.9%) according to the Croatian average. Most patients had completed high school (*n* = 257, 60.9%), and 71.1% (*n* = 300) of the respondents lived with a partner (either married or in a *de facto* relationship).

Breast cancer was the most common type of cancer (*n* = 201, 47.6%), followed by gastrointestinal cancer (*n* = 133, 31.5%). The other tumour sites were less common. The majority of the patients (*n* = 230, 54.5%) were treated for metastatic cancer.

Almost half of the respondents were treated for more than 12 months (*n* = 202, 47.9%), and 59.2% (*n* = 250) were treated with chemotherapy.

Furthermore, 66.4% (*n* = 280) of patients were treated in a GH, and most patients (*n* = 355, 84.1%) lived less than 50 km from the hospital.

### Patients’ perspective on the impact of the COVID-19 pandemic on cancer care

#### Discontinuation of treatment or change in treatment

The results of our study showed that, during the COVID-19 pandemic, 10.2% of patients (*n* = 43) had their oncology treatment discontinued or changed (Tables 2–5).

Among the patients who *discontinued treatment* (*n* = 19, 4.5%), 63% (*n* = 12) discontinued treatment on the recommendation of their oncologist, and 37% (*n* = 7) discontinued treatment themselves. The most common reasons for oncologists to recommend discontinuing treatment were higher risk of COVID-19 (91.7%) and a higher risk of serious complications of COVID-19 (8.3%). The most common reasons why patients decided to discontinue treatment were pandemic fear (28.6%), inability to come to the hospital (57.1%) and self-isolation (14.3%). The majority of the patients (*n* = 18 of 19 patients, 94.7%) were satisfied with the information received from oncologists regarding treatment discontinuation, and 13 (68.4%) of 19 patients felt that the discontinuation of treatment would not affect their disease course or prognosis.

To check for any differences between the patients who discontinued treatment and those who did not discontinue, we performed a chi-square test using sex, age, partnership status, disease site, disease stage and distance to the hospital and a *Fisher’s exact test* using household income and education level (Table 2). Patients were divided into groups according to the disease site: breast cancer, colorectal cancer and others. The ‘others’ category included all other disease sites due to their low frequency.

There was a significant correlation between treatment discontinuation and the distance to the hospital (*p* < 0.05). This indicates that patients who resided more than 50 km from the hospital were more likely to discontinue treatment.

There was no significant correlation between treatment discontinuation and sex (*p* = 0.711), age (*p* = 0.572), household income (*p* = 0.267), educational degree (*p* = 1.00), partnership status (*p* = 0.799), disease site (*p* = 0.355) or disease stage (*p* = 0.438).

In patients who had a *change in treatment* (*n* = 24 of 422 patients, 5.7%), the most common change was in the treatment schedule (58.3%). Other reasons for treatment changes were as follows: change in the type of therapy (29.2%), long waiting time for the start of chemotherapy treatment (4.2%) and delayed surgery (4.2%).

The change in treatment was most often recommended by an oncologist (*n* = 21 of 24 patients, 87.5%) due to a higher risk of COVID-19 (66.7%), higher risk of serious complications of COVID-19 (4.8%), comorbidity (4.8%), side effects of treatment (9.5%), additional tests (4.8%) and inability to come to the hospital (4.8%). Treatment was changed at the request of three patients (*n* = 3 of 24 patients, 12.5%) due to pandemic fear (66.7%) and problems with diagnostic processing (33.3%). All patients who had a change in treatment (*n* = 24, 100%) were satisfied with the information they received from their oncologist, and most patients (*n* = 17 of 24 patients, 70.8%) thought that the change in treatment would not affect the course of their disease and prognosis.

The chi-square test did not show any significant correlation between treatment change and sex (*p* = 0.966), age (less or more than 55 years, *p* = 0.247), partnership status (*p* = 0.664), disease site (breast cancer, colorectal cancer, *p* = 0.762), disease stage (*p* = 0.973) and distance to the hospital (*p* = 0.913)*. The Fisher's exact test* did not show a significant correlation between treatment change and household income (*p* = 0.847), or educational degree (*p* = 0.914; Table 3).

During the COVID-19 pandemic, most patients did not *change their place of treatment* due to the lockdown (*n* = 412, 97.6%). Patients who changed their place of treatment most often (*n* = 9) replaced the treatment at a UH with treatment at a GH/County Hospital on the recommendation of their oncologist. All patients (100%) who changed their place of treatment expressed satisfaction with the oncologists’ communication regarding the change. Most patients (*n* = 9) believed that a change in treatment place would not affect the course of their disease or prognosis.

#### Change in the quality of treatment and diagnostics

Most patients did not experience a change in the *quality of cancer treatment* during the pandemic (*n* = 395, 93.6% of 422 patients). Moreover, some patients (*n* = 19, 4.5%) felt that there was an improvement in the quality of oncology care, probably due to a smaller influx of non-oncology patients into health facilities during the pandemic. In comparison, a smaller number of patients* (n* = 8, 1.9%) considered that the quality of their treatment had deteriorated.

A significant correlation was found between sex, household income and the disease site and changes in oncology treatment quality (*p* < 0.05). Males and low-income patients were more likely to have perceived a change in the quality of their treatment. The quality of treatment was more likely to remain the same in patients with breast cancer. However, a marginally significant correlation was obtained between age and change in treatment quality (*p* = 0.050). We assume that this finding may indicate that a significant difference in the population still exists. This could probably be proven with a larger sample. There was no significant difference between the change in the quality of treatment and education level (*p* < 0.076), partnership status (*p* < 0.661), stage of the disease (*p* = 0.608) or distance to the hospital (*p* = 0.140; Table 4).

In addition to changes in the quality of treatment, we were also interested in whether there was a change in the *quality of diagnostic processing* during the pandemic. Thirty-five patients (8.3%) reported a change in diagnostic quality (Table 5). There was no significant correlation between the change in the quality of diagnostic processing and sex (*p* = 0.353), age (*p* = 0.608), household income (*p* = 0.186), education level (*p* < 0.222), partnership status (*p* < 0.963), disease site (*p* = 0.739), disease stage (*p* = 0.276) or distance to the hospital (*p* = 0.096).

However, we found that 14.7% (*n* = 62) of patients reported a change in the quality of cancer care due to the pandemic.

## Discussion

In the first months of the COVID-19 pandemic, Croatia had a favourable epidemiological situation [8].

Accordingly, 10.2% of patients had an interruption or change in their oncological treatment. In most patients, the reason for this change was on the recommendation of an oncologist owing to the possible higher risk of COVID-19 and a higher risk of serious complications. The most common change in treatment was related to a change in the treatment schedule regardless of clinical or sociodemographic data and the distance to the hospital. But patients who resided more than 50 km away from the hospital were more likely to discontinue treatment regardless of their sociodemographic or clinical data.

A smaller number of patients in our study changed the treatment site due to the lockdown (*n* = 10, 2.4%). They generally replaced the treatment at a University with a County Hospital, which is usually closer to where patients live.

Most of the patients did not experience a change in the quality of cancer treatment during the pandemic (*n* = 395 of 422 patients, 93.6%). Our study showed that, in patients with breast cancer, the quality of treatment was more likely to remain the same. Males and low-income patients were more likely to report a change in treatment quality. 8.3% patients (*n* = 35) reported a change in diagnostic quality regardless of their clinical or sociodemographic data and distance to the hospital. In summary, 62 patients (14.7%) reported a change in the quality of cancer care during the pandemic.

The first study on patients’ perspectives on the influence of the COVID-19 pandemic on cancer treatment, published by Indian authors, showed that cancer patients from India wish to continue chemotherapy during the pandemic and are more worried about cancer progression than the COVID-19 [16]. A study by Dutch authors showed that, in the Netherlands, 30% of cancer patients experienced changes to their oncology treatment or follow-up [17]. The U.S. study showed a 20%–30% reduction in oncology products during the COVID-19 pandemic, while a study by Italian authors confirmed the pandemic’s impact on cancer care, but also that cancer patients are usually strongly motivated to continue cancer treatment despite the pandemic [18, 19].

This study has several strengths. Patients from all counties of Croatia were included. These counties differ in terms of the local epidemiological situation related to COVID-19. Furthermore, the study included 8 of the 13 oncology centres in Croatia, of which two are oncology clinical centres, and six are non-clinical oncology centres, therapy increasing the multicentricity. Furthermore, the respondents were all anonymous.

This study also has several limitations. First, this study did not include patients who did not present at health facilities as planned during the survey period. These patients may not have presented due to objective reasons (issues with transportation due to lockdown, distance from the place of treatment, unable to arrange an escort) or subjective reasons such as pandemic fear. The research was conducted only in the first wave of the COVID-19 pandemic in Croatia, and due to the pandemic of an unknown virus, as well as due to lockdown, the reactions of patients could have been more intense. Although the study included patients from all counties in Croatia, this study did not include the oncology centre of Split-Dalmatia County, where 11% of the population lives [8] and which had the most unfavourable epidemiological situation in Croatia in terms of the number of people infected and the number of deaths.

The COVID-19 pandemic is still ongoing, and despite many efforts, the ideal treatment approach for patients with cancer during the COVID-19 pandemic is still unclear. Due to the overload of health systems, there is a real danger that oncology care will become unavailable in some parts of the world [20].

## Conclusion

In the first few months of the pandemic, Croatia had a favourable epidemiological situation. However, 25% of patients with cancer reported that the pandemic affected cancer treatment and the quality of cancer care.

## Conflicts of interest

All authors have declared no conflicts of interest.

## Funding statement

The authors received no financial support for the research, authorship and/or publication of this article.

## Declaration of authorship

RKD, AB, NDP and IKF conceived and designed the study; RKD, AB, NDP, IKF, TS, VTD, MN, ZC, ZBM, TL, DT, ZV and IT collected, analysed and interpreted data; all authors made the manuscript; RKD, AB, NDP and SP have critically revised the manuscript for important intellectual content; all authors approved the submitted version; all authors agree that they will be responsible for all aspects of the work.

## Figures and Tables

**Table 1.** Demographic and baseline clinical characteristics of the patients.

Characteristic	Patients (*N* = 422)
**Median age (IQR), years**	60.0 (18) 18–82
**Sex**	
Male	139 (32.9)
Female	283 (67.1)
**Household income**	
Low	191 (45.3)
Medium	164 (38.9)
High	67 (15.9)
**Education level**	
Primary education	67 (15.9)
Secondary education	257 (60.9)
Tertiary education	98 (23.2)
**Partnership status**	
In partnership (married or *de facto*)	300 (71.1)
Single, divorced, widowed	122 (28.9)
**Tumour diagnosis**	
Breast cancer	201 (47.6)
Gastrointestinal cancer	133 (31.5)
Genitourinary cancer	30 (7.1)
Lung cancer	22 (5.2)
Other	19 (4.5)
Gynaecology cancer	17 (4.0)
**Tumour stage**	
Non-metastatic cancer	192 (45.5)
Metastatic cancer	230 (54.5)
**Duration of disease**	
1–6 months	133 (31.5)
6–12 months	87 (20.6)
More than 12 months	202 (47.9)
**Types of treatment**	
Chemotherapy	250 (59.2)
Target/immunotherapy	101 (23.9)
Combination of different therapies	71 (16.8)
**Hospital**	
UH	142 (33.6)
GH	280 (66.4)
**Distance to the hospital**	
Less than 50 km	355 (84.1)
50–100 km	33 (7.8)
More than 100 km	34 (8.1)

Data are presented as *n* (%) unless noted otherwise

**Table 2.** Relationship between the discontinuation of treatment and sociodemographic and clinical data (*N* = 19).

Characteristics	*p-*value	
**Sex**		0.711^a^
Male	7 (1.7)	
Female	12 (2.8)	
**Age**		0.572^a^
Less than 55 years	8 (1.9)	
55+ years	11 (2.6)	
**Household income**		0.267^b^
Low	12 (2.8)	
Medium	6 (1.4)	
High	1 (0.2)	
**Education level**		1.00^b^
Elementary	3 (0.7)	
Secondary	12 (2.8)	
Undergraduate and postgraduate	4 (0.9)	
**Partnership status**		0.799^a^
In partnership	14 (3.3)	
Single	5 (1.2)	
**Tumour diagnosis**		0.355^a^
Breast cancer	8 (1.9)	
Colorectal cancer	7 (1.7)	
Other	4 (0.9)	
**Tumour stage**		0.438^a^
Non-metastatic	7 (1.7)	
Metastatic	12 (2.8)	
**Distance to the hospital**		<0.050^a^*
Less than 50 km	12 (2.8)	
More than 50 km	7 (1.7)	

Data are presented as *n* (%) unless noted otherwise

a *p*-value calculated using a chi-square test

b *p*-value calculated using Fisher’s exact test

* A two-sided asymptotic *p*-value of <0.05 was considered to indicate statistical significance

**Table 3.** Relationship between the change in treatment and sociodemographic and clinical data (*N* = 24).

Characteristics	*p*-value	
**Se**x		0.966^a^
Male	8 (1.9)	
Female	16 (3.8)	
**Age**		0.247^a^
Less than 55 years	6 (1.4)	
55+ years	18 (4.3)	
**Household income**		0.847^b^
Low	12 (2.8)	
Medium	8 (1.9)	
High	4 (0.9)	
**Education level**		0.914^b^
Elementary	4 (0.9)	
Secondary	14 (3.3)	
Undergraduate and postgraduate	6 (1.4)	
**Partnership status**		0.664^a^
In partnership	18 (4.3)	
Single	6 (1.4)	
**Tumour diagnosis**		0.762^a^
Breast cancer	10 (2.4)	
Colorectal cancer	7 (1.7)	
Others	7 (1.7)	
**Tumour stage**		0.973^a^
Non-metastatic	11 (2.6)	
Metastatic	13 (3.1)	
**Distance to the hospital**		0.913^a^
Less than 50 km	20 (4.7)	
More than 50 km	4 (0.9)	

Data are presented as *n* (%) unless noted otherwise

a *p*-value calculated using a chi-square test

b *p*-value calculated using Fisher’s exact test

**Table 4.** Relationship between the change in the quality of treatment and sociodemographic and clinical data (*N* = 27).

Characteristic	*p*-value	
Sex		<0.050^a^*
Male	15 (3.6)	
Female	12 (2.8)	
**Age**		0.050^a^
Less than 55 years	5 (1.2)	
55+ years	22 (5.2)	
**Household income**		<0.050^b^**
Low	19 (4.5)	
Medium	5 (1.2)	
High	3 (0.7)	
**Education level**		0.076^b^
Elementary	8 (1.9)	
Secondary	16 (3.8)	
Undergraduate and postgraduate	3 (0.7)	
**Partnership status**		0.661^a^
In partnership	18 (4.3)	
Single	9 (2.1)	
**Tumour diagnosis**		<0.050^a^*
Breast cancer	6 (1.4)	
Colorectal cancer	8 (1.9)	
Others	13 (3.1)	
**Tumour stage**		0.608^a^
Non-metastatic	11 (2.6)	
Metastatic	16 (3.8)	
**Distance to the hospital**		0.140^a^
Less than 50 km	20 (4.7)	
More than 50 km	7 (1.7)	

Data are presented as *n* (%) unless noted otherwise

a *p*-value calculated using a chi-square test

b P-value calculated using Fisher’s exact test

* A two-sided asymptotic *p* < 0.05 was considered to indicate statistical significance

** A two-sided exact *p* < 0.05 was considered to indicate statistical significance

**Table 5.** Relationship between changes in the quality of cancer diagnostic and sociodemographic and clinical data (*N* = 35).

Characteristic	*p*-value	
Sex		0.353^a^
Male	14 (3.3)	
Female	21 (5.0)	
**Age**		0.608^a^
Less than 55 years	14 (3.3)	
55+ years	21 (5.0)	
**Household income**		0.186^a^
Low	16 (3.8)	
Medium	10 (2.4)	
High	9 (2.1)	
**Education level**		0.222^a^
Elementary	3 (0.7)	
Secondary	26 (6.2)	
Undergraduate and postgraduate	6 (1.4)	
**Partnership status**		0.963^a^
In partnership	25 (5.9)	
Single	10 (2.4)	
**Tumour diagnosis**		0.739^a^
Breast cancer	16 (3.8)	
Colorectal cancer	7 (1.7)	
Others	12 (2.8)	
**Tumour stage**		0.276^a^
Non-metastatic	19 (4.5)	
Metastatic	16 (3.8)	
**Distance to the hospital**		0.096^a^
Less than 50 km	26 (6.2)	
More than 50 km	9 (2.1)	

Data are presented as *n* (%) unless noted otherwise

a *p*-value calculated using a chi-square test

^b^*p*-value calculated using Fisher’s exact test
